# Executive Summary of Clinical and Technical Guidelines for Esophageal Cancer Proton Beam Therapy From the Particle Therapy Co-Operative Group Thoracic and Gastrointestinal Subcommittees

**DOI:** 10.3389/fonc.2021.748331

**Published:** 2021-10-19

**Authors:** Michael D. Chuong, Christopher L. Hallemeier, Heng Li, Xiaorong Ronald Zhu, Xiaodong Zhang, Erik J. Tryggestad, Jen Yu, Ming Yang, J. Isabelle Choi, Minglei Kang, Wei Liu, Antje Knopf, Arturs Meijers, Jason K. Molitoris, Smith Apisarnthanarax, Huan Giap, Bradford S. Hoppe, Percy Lee, Joe Y. Chang, Charles B. Simone, Steven H. Lin

**Affiliations:** ^1^ Department of Radiation Oncology, Miami Cancer Institute, Miami, FL, United States; ^2^ Department of Radiation Oncology, Mayo Clinic, Rochester, MN, United States; ^3^ Department of Radiation Oncology, Johns Hopkins University, Baltimore, MD, United States; ^4^ Department of Radiation Oncology, MD Anderson Cancer Center, Houston, TX, United States; ^5^ Department of Radiation Oncology, New York Proton Center, New York, NY, United States; ^6^ Department of Radiation Oncology, Mayo Clinic, Scottsdale, AZ, United States; ^7^ Department of Radiation Oncology, University of Groningen, Groningen, Netherlands; ^8^ Department of Radiation Oncology, University of Maryland, Baltimore, Baltimore, MD, United States; ^9^ Department of Radiation Oncology, University of Washington, Seattle, WA, United States; ^10^ Department of Radiation Oncology, University of Miami, Miami, FL, United States; ^11^ Department of Radiation Oncology, Mayo Clinic, Jacksonville, FL, United States

**Keywords:** esophageal cancer, proton beam therapy (PBT), chemoradiation, pencil beam scanning, passive scatter proton

## Abstract

Radiation therapy (RT) is an integral component of potentially curative management of esophageal cancer (EC). However, RT can cause significant acute and late morbidity due to excess radiation exposure to nearby critical organs, especially the heart and lungs. Sparing these organs from both low and high radiation dose has been demonstrated to achieve clinically meaningful reductions in toxicity and may improve long-term survival. Accruing dosimetry and clinical evidence support the consideration of proton beam therapy (PBT) for the management of EC. There are critical treatment planning and delivery uncertainties that should be considered when treating EC with PBT, especially as there may be substantial motion-related interplay effects. The Particle Therapy Co-operative Group Thoracic and Gastrointestinal Subcommittees jointly developed guidelines regarding patient selection, treatment planning, clinical trials, and future directions of PBT for EC.

## Introduction

Chemoradiotherapy (CRT), delivered either preoperatively or definitively, is critical for the management of locally advanced esophageal cancer (EC) (1, 2). Because of the central anatomic location of the esophagus, organs at risk (OARs) within the chest and upper abdomen receive unintended radiation dose to potentially large volumes when treating with x-ray therapy (XRT), and this may lead to serious acute and/or late toxicities. As such, more conformal XRT techniques like intensity-modulated radiation therapy (IMRT) have been shown to potentially improve clinical outcomes including overall survival (OS) by reducing heart dose and the risk of cardiac death compared to 3D conformal radiation therapy (3DCRT) (3).

For nearly all cases, proton beam therapy (PBT) significantly reduces normal organ dose compared with 3DCRT and IMRT. Proton beams carry charged particles that have relatively low doses in the path proximal to the tumor and deposit most of their energy around the end of its path, called the Bragg peak, the depth of which is determined by the specific energy imparted to the protons, while the OARs beyond the tumor receive essentially no dose. In contrast, the interaction of an x-ray beam within tissue has a relatively superficial dose build-up region and then exponential reduction in dose with increasing depth. Given the excellent conformality of both modern PBT and XRT, the difference in dose to normal tissues is most pronounced at low and moderate levels rather than higher doses at or near prescription dose.

The dosimetric advantages of PBT versus XRT were first demonstrated using the passive scattering (PS) technique, in which apertures and compensators shape the diverging proton beam to achieve appropriate target conformality laterally and distally, respectively. However, a limitation of PS-PBT is reduced conformality proximal to the target. Pencil beam scanning, also commonly referred to as intensity-modulated proton therapy (IMPT), is a modern technique in which “spots” of protons are directed by steering magnets across multiple dose layers, achieving excellent conformality including proximal to the target. Despite these dosimetric advantages, there are a number of PBT planning and delivery uncertainties that should be considered and mitigated using thoughtful treatment planning and delivery techniques.

Prospective and retrospective studies have demonstrated that PBT for EC is well tolerated and clinical benefit may be achieved by significantly reducing normal organ dose (4). With mounting clinical evidence in support of PBT for EC, coupled with an increasing number of PBT centers worldwide, a standardized approach of robust PBT planning and treatment delivery is needed. To meet this growing need, the Particle Therapy Co-Operative Group (PTCOG) Thoracic and Gastrointestinal Subcommittees have jointly generated evidence-based PBT guidelines for EC, highlighting the supporting clinical evidence and recommended treatment planning approaches.

## Dosimetric Advantages of PBT

The central anatomic location of the thoracic esophagus makes PBT particularly attractive for reducing normal organ dose (**Table 1**). In 2008, Zhang et al. were among the first to demonstrate that thoracic OARs could be better spared with PBT as compared to XRT while maintaining excellent target coverage (5). Subsequent comparisons have demonstrated that PBT consistently achieves ≥30%–60% relative reductions in mean heart dose and ≥30%–60% relative reductions in heart V20–V40 compared to IMRT or 3DCRT (6–17). Moreover, PBT achieves ≥40%–60% relative reduction in mean lung dose and ≥30%–50% relative reduction in lung V20. For example, in a study of 55 patients planned to 50.4 Gy in 28 fractions with PS-PBT (typically using a posterior beam and a left lateral beam, weighted 2:1) or IMRT, the PBT plans resulted in significantly lower mean dose to the heart (13.0 vs. 19.9 Gy) and lung (6.3 vs. 9.3 Gy) (8). However, because of the 3D planning approach used for PS-PBT, heart V40 was higher with PS-PBT versus IMRT, owing to the greater conformality index of IMRT.

**Table 1:** Select dosimetric analyses of proton beam therapy versus intensity modulated radiation therapy for esophageal cancer.

Author	Rx dose/Fx	PBT technique	PBT beam arrangement	Heart mean (Gy)			Heart V30			Lung mean (Gy)			Lung V20			Liver mean (Gy)		
				PBT	IMRT	Rel. Δ	PBT	IMRT	Rel. Δ	PBT	IMRT	Rel. Δ	PBT	IMRT	Rel. Δ	PBT	IMRT	Rel. Δ
Zhang et al. (5)	50.4/28	PS	AP/PA 3F	–	–	–	–	–	–	4.5 6.6	9.6	−53.1% −31.3%	9.7% 10.6%	15.6%	−37.8% −32.1%	–	–	–
Welsh et al. (6)	65.8/28	PBS	AP/PA LPO/RPO AP/LPO/RPO	19.9 11.9 17	21.2	−6.1% −43.9% −19.8%	23% 17% 20%	25%	−8% −32% −20%	3.2 4.9 4.3	8.3	−61.4% −41.0% −48.2%	7% 11% 7%	14%	−50.0% −21.4% −50.0%	4.9 5.0 5.4	14.9	−67.1% −66.4% −63.8%
Ling et al. (7)	50.4/28	PS	LAT/LPO	12.6	28.5	−55.8%	20.9%	42.3%	−50.6%	6.0	9.5	−36.8%	15.3%	16.2%	−5.6%	3.6	18.1	−80.1%
Wang et al. (8)	50.4/28	PS	various	13.0	19.2	−32.3%	21.2%	23.7%	−10.5%	6.3	9.3	−32.3%	21.7%	31.4%	−30.9%	3.7	12.4	−70.2%
Shiraishi et al. (9)	50.4/28	PS, PBS	AP/PA; others	13.3	23.7	−43.9%	21.5%	32.3%	−33.4%	–	–	–	–	–	–	–	–	–
Liu et al. (10)	50.4/28	PBS	2-4 beams	7.6	21.9	−65.3%	11.5%	18.5%	−37.8%	3.7	8.6	−57.0%	8.6%	10.3%	−16.5%	2.6	15.3	−83.0%
Makishima et al. (11)	60/30	PS	AP/PA	–	–	–	21.5%	63.3%	−66.0%	5.7	9.3	−38.7%	12.5%	19.4%	−35.6%	–	–	–
Warren et al. (12)	50-62.5/28	PBS	AP/LPO/RPO	12.7	21.2	−40.1%	–	–	–	6.3	13.6	−53.7%	6.6%	15.6%	−57.7%	–	–	–
Xi et al. (13)	50.4/28	PS, PBS	PA/LPO	11.6	19.9	−41.7%	18.9%	24.4%	−22.5%	6.5	10	−35.0%	11.3%	18.4%	−38.6%	–	–	–
Hirano et al. (14)	60/30	PBS	–	11.7	9.4	+24.5%	22.0%	50.7%	−56.6%	5.8	9.4	−38.3%	11.7%	17.8%	−34.3%	–	–	–
Macomber et al. (15)	50.4/28	US, PBS	–	9.6	25.8	−62.8%	–	–	–	–	–	–	–	–	–	–	–	–
Celik et al. (16)	41.4/23	PBS	LPO/RPO AP/LPO/RPO	3.7 4.0	9.9	−62.6% −59.6%	4.9% 4.9%	5.9%	−16.9% −16.9%	3.2 2.9	8.6	−62.8% −66.3%	6.9% 5.9%	10.5%	−34.3% −43.8%	–	–	–

Rx, prescription; PBT, proton beam therapy; PBS, pencil beam scanning; PS, passive scattering; US, uniform scanning; Rel., relative; AP/PA, anteroposterior/posteroanterior; 3F, 3 field; LPO, left posterior oblique; RPO, right posterior oblique; LAT, lateral; Gy, Gray.

IMPT offers improved conformality over PS-PBT with reduction in higher dose to normal tissues. Shiraishi et al. evaluated dosimetric outcomes in 727 EC patients who received PS-PBT (*n* = 237), IMPT (*n* = 13), or IMRT (*n* = 477) (9). IMPT was associated with significantly lower dose to the heart and various cardiac substructures (left atrium, right atrium left main coronary artery, left circumflex artery) compared to PS-PBT.

In addition to heart and lung sparing, PBT also markedly reduces liver dose compared to XRT. In an evaluation of 10 patients with distal EC who were prescribed 50.4 Gy in 28 fractions, the mean liver dose was 3.6 Gy with PS-PBT compared to 18.1 Gy with IMRT (*p* = 0.001) and 20.3 Gy with 3DCRT (*p* = 0.001) (7). Other studies have consistently reported relative mean liver dose reductions of at least 60%–80% and mean liver doses of approximately 5 Gy or less (6, 7, 10).

PBT beam arrangement is an important consideration when evaluating dosimetric differences compared to XRT, especially with respect to the heart and lungs. Welsh et al. evaluated three PBT arrangements (AP/PA, LPO/RPO, and AP/LPO/RPO) and found that the most significant heart sparing was achieved using an LPO/RPO approach (mean: 11.9 *vs*. 21.2 Gy; V30: 17% *vs*. 25%), while much smaller reductions were observed when PBT was planned using AP/PA beams (mean: 19.9 *vs*. 21.2 Gy; V30: 23% *vs*. 25%) (6). On the other hand, PBT plans using AP/PA beams achieved substantially lower lung dose than LPO/RPO plans (mean: 3.2 *vs*. 8.3 Gy; V20: 7% *vs*. 14%). These findings are supported by an analysis from Shiraishi et al. in which certain beam arrangements (especially AP/PA) were associated with high mean heart dose on multivariable linear regression analysis (9). Superior–inferior posterior beams may provide better heart, lung, and liver sparing than LPO/RPO beams (18). Thus, clinical judgment should be used to guide PBT treatment planning with regard to prioritizing OAR sparing and achieving the most clinically appropriate dosimetry for each patient.

## Clinical PBT Outcomes

### Neoadjuvant and Definitive

The published literature describing clinical outcomes of PBT has expanded over the past decade, including both prospective and retrospective evidence that EC patients receive clinically meaningful benefit (**Table 2**) (4, 13, 19, 23, 24).

**Table 2:** Select studies of clinical outcomes comparing proton beam therapy vs. x-ray therapy for esophageal cancer.

Author	No. of patients(RT modality)	Study type	Treatment intent	Follow-up time	Survival	Toxicity or QOL outcomes
Makishima et al. (11)	44 (19 XRT, 25 PBT)	Retrospective cohort	Definitive	NR	N/A	Grade 2+ pulmonary: XRT 18.2%, PBT 0%; Grade 2+ cardiac: XRT 52.6%, PBT 4.0%.
Xi et al. (13)	343 (211 IMRT, 132 PBT)	Retrospective cohort	Definitive	65.1 months	5-year OS: IMRT 31.6% vs. PBT 41.4% (*p* = 0.011); 5-year PFS: IMRT 20.4% vs. PBT 34.9% (*p* = 0.001); 5-year DMFS: IMRT 48.6% vs. PBT 64.9% (*p* = 0.031)	Grade 3–4: IMRT 45.0% vs. PBT 37.9% (*p* = 0.192); Grade 5: IMRT 1.9% vs. PBT 0.8% (*p* = 0.653).
Lin et al. (19)	580 (214 3DCRT, 255 IMRT, 111 PBT)	Retrospective cohort	Neoadjuvant	NR	N/A	Pulmonary complications: 3DCRT 39.5% vs. IMRT 24.3% vs. PBT 16.2% (*p* < 0.001); Cardiac complications: 3DCRT 27.4% vs. IMRT 11.7% vs. PBT 11.7% (*p* < 0.001); Wound complications: 3DCRT 15.3% vs. IMRT 14.1% vs. PBT 4.5% (*p* = 0.014); Mean LOS: 3DCRT 13.2d vs. IMRT 11.6d vs. PBT 9.3d (*p* < 0.0001).
Garant et al. (20)	128 (63 XRT, 62 IMPT)	Prospective registry	Definitive and Neoadjuvant	NR	NR	FACT-E PRO: less mean decline in PRO scores in PBT vs. XRT (−12.7 vs. −20.6, *p* = 0.026).
Routman et al. (21)	144 (65 XRT, 79 PBT)	Retrospective cohort	Definitive and Neoadjuvant	NR	N/A	G4L: XRT 56% vs. PBT 22% (*p* < 0.01).
Davuluri et al. (22)	504 (317 IMRT, 187 PBT)	Retrospective cohort	Definitive and Neoadjuvant	32.1 months	Median OS with or without G4L: 2.8 years vs. 5.0 years (*p* = 0.027); Median PFS with or without G4L: 1.1 years vs. 5.4 years (*p* < 0.001)	G4L: IMRT: 33% vs. PBT 15.5% (*p* < 0.001).
Lin et al. (23)	107 (61 IMRT, 46 PBT)	Prospective phase 2 randomized	Definitive and Neoadjuvant	44.1 months	3-year OS: IMRT 50.8% vs. PBT 51.2% (*p* = 0.60); 5-year PFS: IMRT 44.5% vs. PBT 44.5% (*p* = 0.70)	Mean TTB: IMRT (39.9; 95% highest posterior density interval, 26.2–54.9) vs. PBT (17.4; 10.5–25.0); Mean POC score: IMRT (19.1; 7.3–32.3) vs. PBT (2.5; 0.3–5.2).

3DCRT, three-dimensional conformal radiation therapy; IMRT, intensity modulated radiation therapy; PBT, proton beam therapy; IMPT, intensity-modulated proton therapy; NR, not reported; OR, odds ratio; XRT, X-ray (photon) radiation therapy; LOS, length of hospital stay; OS, overall survival; PFS, progression-free survival; DMFS, distant metastatic-free survival; G4L, grade 4 lymphopenia; FACT-E PRO, Functional Assessment of Cancer Therapy-Esophagus Patient Reported Outcomes; TTB, total toxicity burden; POC, postoperative complication.

In a retrospective study compiling data from three institutions comparing neoadjuvant 3DCRT, IMRT, or PBT with concurrent chemotherapy before esophagectomy, PBT was associated with lower rates of pulmonary and wound healing complications (19). Length of hospitalization was significantly reduced in the PBT group as compared to the XRT cohort, likely a result of reduced postoperative complications. While acute cardiac events were greater in the 3DCRT group, there were no differences between PBT and IMRT. A recent study analyzed cardiovascular events in 479 EC patients treated using IMRT or PBT, in which 18% of patients developed major grade 3+ cardiovascular events with a median follow up of 76 months. Cardiovascular events occurred at a median of 7 months after CRT, the majority of which (81%) occurred within the first 2 years after completing CRT (24). The strongest factors associated with increased risk of grade 3+ events were pre-existing cardiovascular disease and the use of IMRT (*vs*. PBT). Among patients with pre-existing heart disease, the use of PBT was associated with a significantly lower event rate at 2 years compared to IMRT (11 *vs*. 30%; *p* = 0.0018). In addition, a prospective registry study of 125 patients with EC receiving CRT, patients receiving PBS-PT (*vs*. IMRT) had better preservation of health-related quality of life as assessed by the Functional Assessment of Cancer Therapy-Esophagus (FACT-E) questionnaire during CRT (20).

PBT is expected to deliver radiation dose to a lower volume of the total blood pool compared to XRT, and because of the exquisite radiosensitivity of circulating lymphocytes, this difference may be clinically significant (25, 26). For example, a propensity-matched analysis by Shiraishi and colleagues for 480 EC patients demonstrated a markedly higher incidence of grade 4 lymphopenia among patients receiving IMRT compared to PBT (40.4% *vs*. 17.6%; *p* < 0.0001) (27). Investigators from the Mayo Clinic more recently presented similar findings showing a strong association between reduced severe lymphopenia with PBT (28).

Recently, investigators at MD Anderson Cancer Center published results of a phase 2 randomized trial conducted comparing PBT (80% PS-PBT, 20% IMPT) and IMRT (29). The study co-primary endpoints were progression-free survival (PFS) and a novel endpoint called total toxicity burden (TTB), which is a composite index of 11 distinct toxicities of varying grades from the start of CRT up to 1 year. Although the study closed early with 107 patients, it showed a significant improvement in the overall TTB score for PBT by 2.5-fold and a reduction in TTB by 7.6-fold for postoperative complications, without a difference in PFS or OS. This is the first proton versus photon randomized trial across all disease sites with a positive primary endpoint favoring PBT (30). NRG-GI006 (NCT03801876) is a phase 3 randomized controlled trial comparing PBT and IMRT for EC that is currently enrolling patients with the hypothesis that dosimetric advantages of PBT will translate into meaningful clinical benefit. Other prospective clinical trials are ongoing as summarized in **Table 3**.

**Table 3:** Clinical trials of proton beam therapy for esophageal cancer.

NCT ID	Title	Phase	Status	Outcome Measures	Institution
02213497	Dose Escalation of Neoadjuvant Proton Beam Radiotherapy with Concurrent Chemotherapy in Locally Advanced Esophageal Cancer	I	Recruiting	Adverse events	Abramson Cancer Center, University of Pennsylvania
02452021	Pencil Beam Scanning Proton Radiotherapy for Esophageal Cancer	—	Active, not recruiting	Toxicity, surgical outcomes, post- operative complications, LOS, LRR, PFS, OS, QOL	Mayo Clinic
03482791	Proton Beam Therapy in the Treatment of Esophageal Cancer	II	Recruiting	Patient-reported outcomes, PFS, OS	Washington University School of Medicine
01512589	Proton Beam Therapy vs. Intensity-Modulated Radiation Therapy	II	Active, not recruiting	PFS, TTB	The University of Texas MD Anderson Cancer Center
01684904	Proton Therapy for Esophageal Cancer	II	Recruiting	OS, adverse events	Loma Linda University Medical Center
02023541	Proton Beam Therapy to Treat Esophageal Cancer	I	Terminated	PFS, OS, QOL, toxicity	Washington University School of Medicine
03801876	Comparing Proton Therapy to Photon Radiation Therapy for Esophageal Cancer	III	Recruiting	OS, toxicity, pathologic response rate, lymphocyte counts, LRF, DMFS, PFS, QALY, cost–benefit economic analysis	Multicenter

NCT, National Clinical Trials; LOS, length of [inpatient] stay; LRR, local-regional recurrence; PFS, progression-free survival; OS, overall survival, QOL, quality of life; TTB, total toxicity burden; LRF, local-regional failure; DMFS, distant metastasis-free survival; QALY, quality-adjusted life years.

### Reirradiation

For patients with recurrent or *de novo* EC occurring in the context of prior thoracic RT reirradiation can be considered, although the risks of severe late adverse effects can be significant (31). PBT is expected to achieve a potentially large reduction in cumulative doses to critical OARs, including the spinal cord, heart, lungs, proximal bronchial tree, and liver (32–34). Several cohort studies have demonstrated feasibility and encouraging early clinical outcomes with PBT reirradiation for EC (**Table 4**) (31, 33, 35, 36). Most recently, DeCesaris et al. reported outcomes in a retrospective cohort of 37 patients treated with PBT reirradiation at four institutions with a median reirradiation dose of 50.4 Gy and median cumulative dose of 104 Gy (37). Most patients (90%) received concurrent chemotherapy. With a median follow-up of 20 months after reirradiation, the 18-month OS was 56% and the 18-month locoregional control rate was 69%. Late grade 3 toxicity was observed in 24% consisting of strictures/stenosis requiring dilation, while some patients experienced late grade 4–5 toxicities (19%).

**Table 4:** Select studies of proton beam therapy reirradiation for cancers of the esophagus.

Author	Number of patients	Prior RT dose (median)	Cumulative RT dose (median)	Median time to reirradiation	Non-RT treatments	Median follow-up	Disease-control outcomes	Survival outcomes	Toxicity outcomes
Fernandes et al. (27)	14	54 Gy (range 25.5–70 Gy)	109.8 Gy (range 76-129.4 Gy)	32 months (range 10–307 months)	Concurrent chemotherapy (*n* = 11, 79%)	10 months (range 2–25 months)	9/14 (64%) with LRR, 6/14 (43%) with DM, 8/10 (80%) with improved/stable dysphagia	Median OS 14 months (95% CI, 7–21 months), 1-year OS 71%.	Acute: grade 3: dehydration (*n* = 2), dysphagia (*n* = 2), GI bleed (*n* = 1), hyponatremia (*n* = 1), pneumonia (*n* = 1), weight loss (*n* = 1); grade 5: esophagopleural fistula (*n* = 1). Late: grade 3: dysphagia (*n* = 1), esophageal stenosis (*n* = 1), esophageal ulcer (*n* = 1), heart failure (*n* = 1); grade 5: esophageal ulcer (*n* = 1)
DeCesaris et al. (29)	17	53.4 Gy (range 40–108 Gy)	104.7 Gy (range 94-156 Gy)	37.6 months (range 11.6–584 months)	Concurrent chemotherapy (*n* = 15, 88%); chemotherapy preceding RT (*n* = 1,6%)	11.6 months (range 2.0–36.6 months)	1-year LC 75.3%; 1-year DC 83.4%.	Median OS 19.5 months (95% CI, 5.7–33.3 months)	Acute: grade 3: dysphagia (*n* = 1), esophagitis (*n* = 1). Late: grade 3: esophageal stenosis (*n* = 2); grade 4: esophageal stenosis (*n* = 1), TEF (*n* = 1); grade 5: TEF (*n* = 1).
Patel et al. (31)	3	36 Gy (range 15–36 Gy)	NR	30 years (range 5–41 years)	Concurrent chemotherapy (*n* = 3, 100%); post-PBT esophagectomy (*n* = 3, 100%)	26 months (range 22–72 months)	0/3 (0%) with LRR or DM	3/3 (100%) alive at 22, 26, and 72 months post-op	Acute: mild/moderate odynophagia (*n* = 2), esophageal stricture (*n* = 1), hematemesis (*n* = 1), moderate/severe esophagitis (*n* = 1). Late: intra-op cardiac arrest (*n* = 1)

RT, radiation therapy; PBT, proton beam therapy; EC, esophageal cancer; SCC, squamous cell carcinoma; Gy, Gray; GyE, Gray equivalent; PS, passive scatter; PBS, pencil beam scanning; LRR, locoregional recurrence; DM, distant metastasis; OS, overall survival; CI, confidence interval; GI, gastrointestinal; DLBCL, diffuse large B-cell lymphoma; HL, Hodgkin’s lymphoma; LC, local control; DC, distant control; NR, not reported.

## Patient Selection for PBT

Patients with cervical esophagus, thoracic esophagus (upper, middle, or lower), and gastroesophageal junction cancers may be considered to receive PBT. PBT is expected to offer patients clinical benefit when used preoperatively, definitively, or postoperatively.

PBT should be most strongly considered in the following situations:

- Treatment is delivered with curative intent, where greater benefit from mitigation of late toxicity is expected compared to patients treated with palliative intent.
- Patients who have severe medical comorbidities, especially cardiac and/or pulmonary, because of superior heart and lung sparing compared to XRT.
- While patients of all ages are likely to benefit from a lower risk of significant late toxicities with PBT versus XRT, elderly patients who are often at higher risk of treatment-related morbidity and postoperative complications may especially benefit from the superior OAR sparing of PBT (38).
- For patients with local and/or regional recurrence of EC, or newly diagnosed EC arising in a previously irradiated region, PBT should be strongly considered over IMRT especially if treatment intent is curative (31). The American Society for Radiation Therapy (ASTRO) Model Policy for PBT considers re-irradiation (where cumulative critical structure dose would exceed tolerance dose) to be a “Group 1 indication” in which PBT is considered “medically necessary” (39). Care should be taken to evaluate composite dose in an attempt to mitigate the risks of severe toxicity including fistula and hemoptysis, as well as grade 5 events.
- PBT should be considered when dose escalation is used because it may mitigate higher risks of toxicities to critical OARs (40).

PBT may be reasonable although it *should be used cautiously* in the following situations:

- Extensive tumor involvement of the gastric cardia/body (tumor extending ≥5 cm distal to the gastroesophageal junction) may cause potentially sizeable inter- and intra-fractional differences in tumor position resulting in potential geometric miss.
- Variability in stomach filling (air versus fluid) and respiratory motion causing interplay effects should be mitigated, especially when using IMPT (41).
- Use of PBT for patients with pacemakers is considered a relative contraindication, especially for those who are pacemaker dependent, due to concern about neutron dose to the device and risk of subsequent device malfunctioning (42). However, such patients may be treated safely in the context of a well-defined plan for monitoring device function and responding to potential device dysfunction during the course of treatment. Such a plan requires close collaboration with colleagues in cardiology, and preferential use IMPT (*vs*. PS-PBT) if possible due to lower neutron dose.

## Treatment Planning

### Simulation

Prior to simulation, the physician, physicist, dosimetrist, and simulation therapists should discuss the anticipated treatment volume, immobilization technique, and consideration of internal target motion due to respiration and gastric distention. General guidelines include instructing the patient to have a relatively empty stomach before simulation/treatment (if possible) to limit variability in gastric filling and distention. This can be achieved by patients having nothing by mouth (NPO) 2–3 h prior to simulation and treatment in addition to avoiding foods that may cause excess gas. It is preferred to not use oral contrast to minimize stomach distention, and gastrointestinal luminal structures are often well visualized without contrast agents. If oral contrast is used, then a non-contrast-enhanced CT scan should first be performed for treatment planning as contrast material has a significant impact on calculating proton beam range; a second CT scan would then be obtained after contrast is administered, which would serve as a secondary scan for target delineation purposes.

In most situations, the preferred treatment position is head first, supine, and with the arms up above the head in a custom immobilization device. There are some situations in which arms may be placed at the patient’s side, such as patient intolerance of an “arms up” position or a tumor in the cervical or upper thoracic esophagus in which a thermoplastic head and shoulder immobilization device may be preferred. An “arms down” position is a reasonable alternative for patients treated with PBS, as the typical beam arrangement of posterior/posterior oblique fields avoids the arms. However, if there is gastric extension of the tumor (in which the target volume will extend significantly left of midline), the left arm may be in the path of a left posterior oblique beam. Additional potential technical issues created with the “arms down” position, especially for larger patients, include CT beam hardening artifacts from the arms and difficulty including the entire external body surface within the scan field of view. A full body immobilization device or pad under the patient’s back may be considered to improve setup reproducibility and/or patient comfort, although attention should be paid to potential uncertainty in proton stopping power along the beam path. Immobilization of the hips and lower extremities may help facilitate reproducible alignment of the spine.

A non-contrast, free-breathing four-dimensional CT (4DCT) scan should be acquired for treatment planning. Patients may be treated with a free breathing, internal gross tumor volume (iGTV) approach, assuming appropriate motion-robust planning methods are used, as outlined in the next section. Using a breath hold technique may be appropriate for some patients, and if this is to be used, multiple breath hold scans should be acquired to ensure reproducibility of this technique. Some centers have utilized treatment under mechanical ventilation to control breathing variations and reduce the breathing amplitude. In this case, 4DCT imaging should be performed for the same mechanical ventilation situation as intended for treatment. The scan volume should encompass the entire external body surface and immobilization device in the *x-* and *y-*planes and should include the entire lungs and kidneys in the *z-*plane for dose reporting to these organs. If the upper mediastinum and cervical lymph nodes are to be treated, the scan should also include the full neck to the skull base. The CT scan/reconstruction slice thickness should be ≤3 mm. Intravenous (IV) contrast may be administered to aid in target delineation, although this should be done after acquisition of a non-contrast CT for planning/dose calculation.

### Target Delineation

Normal tissue and target delineation should be performed on the non-contrast 4DCT data set. Typically, the CT average series will be utilized for segmentation and planning, as this best represents the time-averaged tissue densities and proton stopping power ratio, especially in the region of the diaphragm. Alternatively, the maximum exhalation series (diaphragm at its most cranial position) may be utilized. The planning scan should be registered with the diagnostic positron emission tomography (PET)/CT to aid in target delineation. Additionally, the esophagogastroduodenoscopy (EGD) report(s) should be reviewed for correlation of tumor extent with imaging studies.

Target delineation for the management of EC is similar whether using photons or protons and should be performed according to published guidelines (43). The gross tumor volume (GTV) should include the primary tumor (GTVp) and involved lymph nodes (GTVn), based on the planning CT, PET/CT, and EGD reports. An iGTV should be contoured, if treatment will be with free breathing, including the GTV on all respiratory phases of the 4DCT or using maximal intensity projection (MIP) images and edited through phases. The clinical target volume (CTV) typically includes up to a 3- to 4-cm expansion of the GTVp along the proximal and distal esophagus/stomach to cover potential microscopic mucosal and submucosal spread. This volume is typically further expanded by 1–1.5 cm radially from the esophagus/stomach to cover potential periesophageal and perigastric lymphatic spread, excluding uninvolved OARs like the heart, lungs, and spine. The CTV also includes a 0.5- to 1-cm expansion of the GTVn, excluding uninvolved OARs. Elective lymph node basins (celiac, gastrohepatic, para-aortic for lower esophagus, and GEJ tumors; supraclavicular for upper esophagus tumors) are typically included in the CTV. The CTV may be further modified based on the 4DCT, generating an internal target volume (ITV) to account for respiratory motion.

Typical prescribed doses are 41.4–50.4 Gy (RBE 1.1) in 23–28 fractions of 1.8–2 Gy for preoperative treatment, 50–50.4 Gy (RBE 1.1) in 25–28 fractions of 1.8–2 Gy for definitive treatment, and 50–60 Gy (RBE 1.1) in 25–30 fractions of 1.8–2 Gy for postoperative treatment. The prescription of higher doses is used at some institutions, especially outside of North America, although this is controversial since no randomized data have demonstrated a clinical benefit for dose escalation (44).

Dose painting techniques may be utilized that deliver differential daily dose to separate volumes. For example, some institutions administer 25 fractions, with a dose of 50 Gy (2 Gy/fraction) to the iGTV + 1 cm margin and a dose of 45 Gy (1.8 Gy/fraction) to the typical CTV described above.

## Passive Scattering Treatment Planning

### Beam Angle Selections

Zhang et al. performed a comparative planning study of two-beam PSPT (AP/PA), three-beam PSPT (AP/LPO/RPO), and IMRT for distal esophageal or GEJ cancer (5). While lung sparing was improved using an AP/PA beam arrangement, there was a trade-off with increased heart dose. As such, preferring posterior-oriented beams should be considered to reduce heart dose. Given the increasing clinical evidence that minimizing heart dose should be prioritized, the LPO/RPO beam arrangement has been adopted as the standard clinical class solution for PSPT plan design at many centers, which is believed to strike an appropriate balance between lung and heart sparing compared to AP/PA and 3-beam PSPT approaches.

### Planning Parameters Selections

Major planning parameters for PSPT include aperture margins, distal margins, proximal margins, smearing margins, and border smoothing margins, and should be chosen for each individual beam once the beam angles are decided for a given treatment plan. The beam-specific target can be created using the target (ICTV) expanded with distal and proximal margin in the beam direction determined by range uncertainty and lateral margin due to setup uncertainty (ICTV to PTV expansion margin, typically 5 mm), as opposed to using a PTV for forward planning (45). Zhang et al. discussed the choice of these planning parameters, based on the method suggested by Moyers et al. (5, 46). Zhang et al. used an aperture margin to ensure that all the proton beams had at least 95% of the PTV receiving the prescription dose, whereas the distal and proximal were 3.5% of the distal and proximal range of the spread-out Bragg peak (SOBP) plus an additional 2–3 mm for other uncertainties. The smearing margin accounts for both the setup error and proton scatter to ensure distal coverage if there is setup error. The value of the smearing margin could initially use the methods described by Moyers et al. and then be adjusted to ensure the beam specific target is covered in average, maximum inhale phase (T0) and patient-specific maximum exhale phase (TExp).

### Plan Evaluation

After the treatment plan is designed based on the average CT, the treatment plan should be recalculated and evaluated on the T0 and TExp of the CT image data set. Diaphragm density overrides, which have been adopted from XRT, can be considered although may not be necessary in some patients to achieve planning and inter-fractional robustness. Although a PTV is only used for the lateral margins of the treatment plan design, the PTV should be used for the evaluation following the recommendation of ICRU 78 (47). The planning parameters including distal margin, proximal margin, and smearing and aperture margins should be adjusted to ensure proper coverage for GTV, ICTV, and PTV on average, T0, and TExp CTs (48).

## Pencil Beam Scanning Treatment Planning

### Beam Angle Selection

Yu et al. reported a water equivalent thickness (WET)-based method to select IMPT beam angles that are robust to respiratory motion for EC treatment, and in specific, diaphragmatic motion (49). Motion robust beam angles were determined by examining the change of WET along the beam path required to cover the target during a full cycle of free-breathing motion at various angles. The beam angles that yielded the smallest value of the maximum temporal change of WET were considered to be the most robust to respiratory motion. The most motion robust beam angles are generally posterior with a median gantry angle of 200°C (range, 180°C–220°C) and couch at 0°C, because these beam angles pass through a relatively less mobile portion of the diaphragm. This choice of beam angles is also optimal from the point of view of avoiding organs with variable filling proximal to the target, such as the stomach. Another consideration could be superior–inferior posterior oblique beams with the couch at 270°C, which may provide better sparing of normal organs lateral to the target at the expense of delivering higher dose to the spinal cord (18). Accordingly, most centers treating distal esophagus/GEJ tumors with IMPT have used two to three posterior oriented beams (18, 20, 33, 50). For treatment of tumors in the cervical and proximal thoracic esophagus, an anterior beam should be considered to reduce lung dose.

### Treatment Plan Design and Evaluation

Various PBT planning strategies can effectively mitigate the effect of respiratory motion and achieve robust plans. For example, use of larger spot size and rescanning/repainting could reduce the effect of motion (51). For distal/GEJ tumors, the diaphragm is usually not part of the target, although it is likely to traverse in and out of the treatment field and could cause significant interplay effects on dose delivery if not properly taken into account. With motion robust beam angles, a high-quality PBS plan can be generated using a single-field optimization (SFO) technique for most EC patients (49). Similar to the PS-PBT plan design, beam-specific targets could be designed for each beam for SFO treatment planning. The optimization algorithm should ensure that the beam-specific target has the adequate coverage for each beam. Yu et al. suggested that a multi-field optimization (MFO) technique could result in a more conformal plan compared to SFO, but the MFO technique is more sensitive to setup, range, and motion uncertainties. Regardless, robust optimization can achieve robust SFO and MFO plans (10, 52, 53). Planning criteria that include appropriate range uncertainties, along with setup uncertainties compatible to the PTV margins, should be applied to CTV dose coverage objectives. Robust optimization can also be applied on high-risk OARs such as the spinal cord, using the same robustness criteria, if such OARs are expected to receive near their maximum tolerance doses. If the plan involves MFO and/or split target volumes, care must be taken to assure that appropriate dose fall-off gradients are introduced in the field or target sub-volume junction regions to assure junction line dose homogeneity in the presence of setup errors and respiratory motion. In addition, a 4D optimization technique (54), for example, where the IMPT plan is optimized to meet dose constraint on multiple 4DCT phases (55), could be used to create motion robust treatment plans. Regardless of optimization technique that was used for plan design, it is important to perform robustness evaluation with regard to setup, range and motion (52). A commonly used technique for setup and range robustness evaluation is the worst-case evaluation where different scenarios include shifted isocenter of the plan on all cardinal directions, and the beam range modified with assumed max range uncertainty. Dose is then calculated and evaluated on all scenarios to identify the worst case for all plans or all voxels in the patient. Additionally, motion robustness could be evaluated using the recalculated dose on the 4D CT scans at T0 and TExp with the original plan (56). Dose on the robustness evaluation plans should meet the planning criteria for GTV, CTV, and OARs as defined on individual data sets. Yu et al. demonstrated that D95 variation between the nominal dose calculated on the average CT and the dose distribution on T0/TExp verification plans highly correlates with D95 variation between the nominal dose and the full 4D dose calculation (49). These results indicated that dose impact of respiratory motion could be evaluated using verifications on T0 and TExp. Ideally, for a comprehensive robustness analysis, all uncertainty components should be evaluated in combination as suggested by Ribeiro et al. (57).

**Figure 1:**
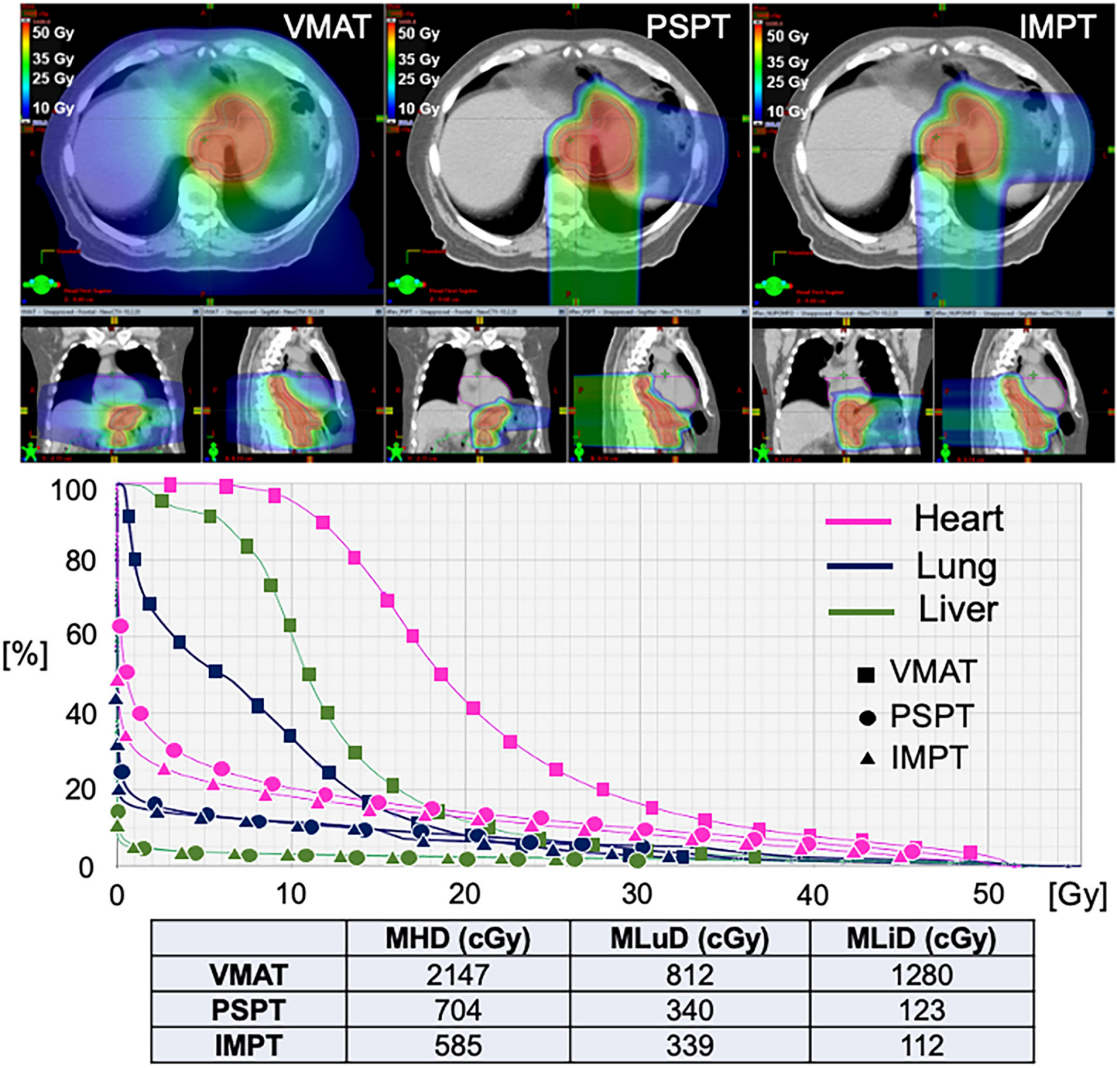
Dosimetric comparison of treatment plans using all three modalities for a patient with distal esophageal cancer. VMAT, Volumetric Arc Therapy; PSPT, Passive Scattering Proton Therapy; IMPT, Intensity Modulated Proton Therapy; MHD, Mean Heart Dose; MLuD, Mean Lung Dose; MLiD, Mean Liver Dose.

**Figure 1** shows a comparison between IMRT, PS-PBT, and IMPT plans for a distal EC patient. The PS-PBT plan uses two PA/LPO, the IMPT plan uses three posterior oblique beams, whereas IMRT uses two arcs. It can be observed that both PS-PBT and IMPT plans better spare OARs including lung, heart, and liver, while maintaining target coverage. In addition, IMPT achieves better conformity of the target and lower mean dose to the lungs, compared to PS-PBT.

## Robust Treatment Planning, Delivery, and Motion Management

The motion management strategy for EC should be similar to those used for treating lung cancers (41). Here, we focus on special considerations of motion management for EC, namely, the impact of respiratory motion.

For treatment planning, the following should be considered:

1. Select robust beam angles that yield the smallest value of the maximum temporal WET change, typically posterior-oriented beams.
2. Adopt a robust optimization strategy. 4D robust optimization is recommended to design PBS treatment plans because it takes density changes along the beam path into consideration.
3. Perform motion evaluation to quantify the geometry motion and voxel WET changes. Diaphragm breathing amplitudes and off-sets can be used as EC motion surrogates to some extent (58). However, to evaluate EC motion in more detail, considering also differential motion, more sophisticated methods (for example, based on deformation vector fields) have to be developed. Further work is required to establish motion mitigation guidelines based on concrete motion limits advising on planning and delivery strategies as, for example, the use of larger spot size (e.g., with range shifters), breath hold, mechanical ventilation, abdominal compression, robust planning, and re-scanning.

For treatment delivery, the following strategies are recommended:

1. For IMPT, use rescanning (either layered or volumetric) to reduce interplay effects.
2. For IMPT, use an optimized delivery sequence, including scanning direction and breath sampling, to minimize interplay effects (52, 59).
3. Use breath hold, mechanical ventilation, abdominal compression, or gating techniques if other strategies are not sufficient to reduce the interplay effect.
4. Use daily image guidance with at least kilovoltage (kV) x-ray imaging. It is strongly recommended that cone beam CT (CBCT), if available, be used at least once weekly to ensure appropriate soft tissue reproducibility.
5. Perform routine quality assurance (QA)/verification CT scans to determine whether adaptive replanning in order to ensure robustness (52). It is recommended that such scans be done at least once during the first 2 weeks of treatment, and then again during the third or fourth week, recognizing that this is subject to patient- and center-specific factors.

## Quality Assurance and Adaptive Replanning

Similar to other disease sites, patient-specific QA for EC patients includes various components, plan evaluation, robustness analysis with respect to range and setup uncertainties and motion interplay effect, and measurements in phantoms. Measurements of dose distributions of individual fields for IMPT are challenging because of the complex dose distributions of the treatment fields. Measurements are commonly made with a two-dimensional ion chamber array detector, and 3D detectors are being developed (60). Like other sites in thorax and abdomen, motion interplay effects and the effectiveness of motion mitigation strategies for EC patients should be considered as part of the patient QA process, although they remain in development (21). From treatment planning, one can evaluate the verification plans on the T0 and TExp phases of the 4DCT image data set to estimate systematic errors caused by motion interplay effect. Ideally, one should use a 4D phantom to measure the dose distribution to assess the motion interplay effect. This should be done during commissioning if not every patient. However, the challenge is the lack of any commercially available standardized 4D phantom. Recently, efforts have been made to optimize QA workflows. Efforts such as those described by Meijers et al. basing patient specific quality assurance on independent dose calculation and predicted outcomes should find broader application in the future (61).

To assess the impact of variation in anatomy and tumor size, patients should undergo repeat 4D CT verification scans to determine whether offline adaptive replanning is needed to maintain target coverage (e.g., >95% for the ICTV) and to avoid overdosing critical structures (52). 4D magnetic resonance imaging has become available for motion verification, with advantages over CT being superior soft tissue contrast, no imaging dose, and longer data sampling interval. In-room volumetric imaging techniques such as CBCT and in-room CT could also be used to identify possible anatomy changes. However, dose calculation on CBCT may not be sufficiently accurate. Therefore, verification 4D CT scans should be performed several times during the course of PBT to generate verification plans. If the patient is treated with breath hold technique, the verification CT scans should be performed using similar breath hold technique to verify consistency of diaphragm position and shape and resultant impact on dose distribution. 4D dose reconstruction and accumulation based on available repeated 4D images enables the clinical estimation of actual exhibited interplay and motion effects, facilitating an informed triggering of adaptive replanning (21). If an adaptive plan is deemed necessary, then it should be developed by using techniques as described earlier, and the same patient-specific quality assurance (PSQA) process should be repeated before treatment with the adapted plan. Similarly, if a phase-based gating strategy is utilized for treatment, then the same phases should be used for the verification plan. On the verification scans, careful attention should be paid to potential changes in external anatomy (from setup, immobilization, weight loss or gain), internal anatomy (tumor shrinkage or swelling, change in esophagus/stomach filling, presence/absence of an esophageal stent, presence/absence of pleural effusion, change in diaphragm position/shape), and motion pattern, which could require further systematic monitoring and timely adjustment of treatment plans. Adaptive PBT has the ability to correct for dosimetric effects induced by interfractional anatomic changes and it complements the ability of image guided setup to correct for setup uncertainty.

## Future Directions

With increasing awareness that the immune system plays an integral role in cancer-related outcomes, attention should be directed at mitigating radiation-related effects especially on lymphocytes that are radiosensitive even to doses as low as <1 Gy (22). High-grade lymphopenia has been associated with poorer long-term outcomes among EC patients (25) and may be significantly reduced using PBT as compared to photon radiotherapy (26–28). Future consideration should be given to the development of lymphopenia-related organ-at-risk constraints for the heart, lungs, major vascular structures, bone marrow, and spleen that would be routinely incorporated into treatment plan optimization. The importance of mitigating RT-related severe lymphopenia, and thus the benefits or PBT, may become even greater should immune checkpoint inhibitors become standardly delivered for localized EC.

Although photon-based dose escalation studies have been negative for EC (44, 62), this concept could be re-explored in the context of reduced OAR dose afforded by PBT (63). As standard doses utilized for EC management lead to suboptimal rates of locoregional failures, employing proton therapy to better protect adjacent critical structures from unnecessary irradiation and resulting treatment-related morbidity and mortality may more safely allow for dose escalation (62), and a potential improvement in tumor control without added toxicity. This may be particularly important in patients managed without surgery with definitive radiation therapy.

**Figure 2:**
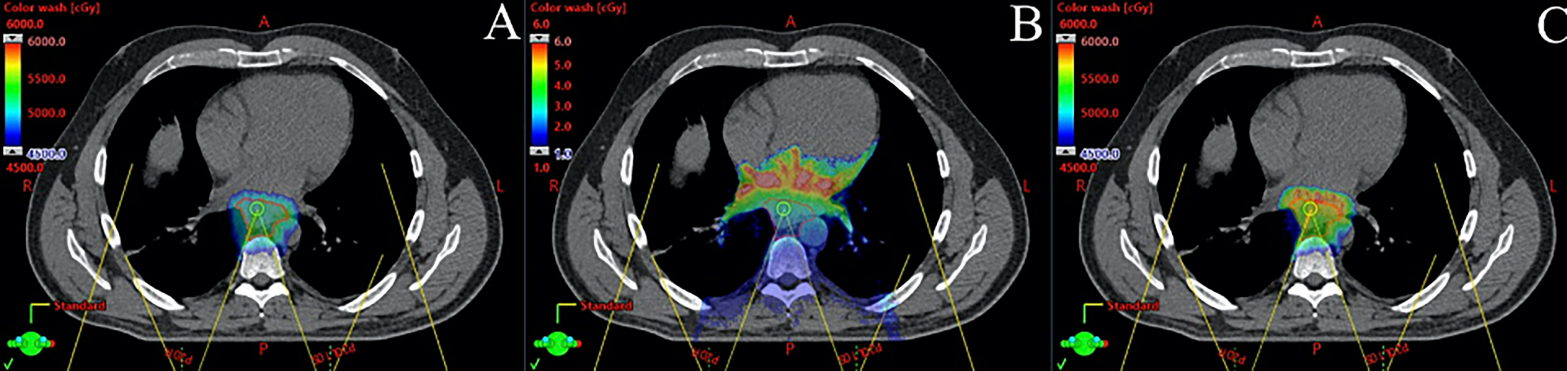
Monte Carlo dose calculation for a patient with distal esophageal cancer treated with 5,000 cGy in 25 fractions using pencil beam scanning proton beam therapy with two posterior oblique fields. **(A)** shows physical dose in cGy, **(B)** shows linear energy transfer (LET) in keV/μm, and **(C)** shows modeled biological dose incorporating physical dose and LET. Note that the high LET region is distal to the target in the heart.

The biologic effects of PBT within tumor and OARs are not well understood and warrant further study. Proton beams have a modestly higher radiobiological effectiveness (RBE) compared to photon beams; therefore, a correction factor of 1.1 is commonly applied to the absorbed dose. However, it is recognized that the RBE is variable over the depth dose curve, namely, it is higher in the region of the Bragg peak, and especially the distal end of the Bragg peak, due to higher LET (64). Because of this, PBT treatment plans have traditionally avoided beams that “range out” into a critical structure for fear of biologic dose enhancement. This may have implications for use of PBT for EC, and especially for PBS techniques, which utilize posterior beams that may “range out” into critical structures including the heart, stomach, and intestine. The clinical effects of this are currently unknown. Sophisticated planning techniques are being developed that allow one to visualize the location of high LET, which could be considered in the planning process and plan optimization that has the potential to further reduce toxicities and also improve tumor control with LET-based planning (**Figure 2**) (65–67).

Lastly, because interplay effects especially with IMPT can significantly degrade dose distribution in the thorax (67), a PSQA process or similar approaches should routinely consider such effects (10). For IMPT, this can be done by simulating the temporal relationship between the time-dependent spot delivery and respiratory motion. Commerical treatment planning systems are expected to offer interplay effect evaluation in the future.

## Conclusions

PBT should be strongly considered for trimodality and non-operative thoracic EC patients based on retrospective and randomized prospective data that demonstrate clinically meaningful reductions in toxicity compared to XRT. Robust PBT plan development and treatment delivery is critical to ensuring appropriate target and surrounding OAR dosimetry. Long-term toxicity and efficacy outcomes of PBT versus XRT are being evaluated in the ongoing NRG-GI006 phase 3 randomized trial (NCT03801876), and we encourage enrollment on that study.

## Author Contributions

MC, CH, CS, and SL contributed to the conception and design of the manuscript. All authors contributed to the article and approved the submitted version.

## Conflict of Interest

The authors declare that the research was conducted in the absence of any commercial or financial relationships that could be construed as a potential conflict of interest.

## Publisher’s Note

All claims expressed in this article are solely those of the authors and do not necessarily represent those of their affiliated organizations, or those of the publisher, the editors and the reviewers. Any product that may be evaluated in this article, or claim that may be made by its manufacturer, is not guaranteed or endorsed by the publisher.
